# A Case of Pityriasis Lichenoides Chronica Successfully Treated With Upadacitinib

**DOI:** 10.1155/crdm/1118647

**Published:** 2026-04-06

**Authors:** Olivia R. Noble, David J. Cohen, Lynn Cooper

**Affiliations:** ^1^ Department of Medicine, Medical College of Georgia at Augusta University, Augusta, Georgia, USA, augusta.edu; ^2^ Skin Care Physicians of Georgia, Macon, Georgia, USA; ^3^ Skin Cancer Specialists, P.C., Marietta, Georgia, USA

## Abstract

Pityriasis lichenoides chronica (PLC) is a chronic cutaneous disorder of unknown cause that is thought to be a proliferation of T‐cells in response to an antigen or infection. We introduce a case of a 62‐year‐old female who presented with an erythematous nonpainful eruption of well‐demarcated annular and polycyclic papules and plaques with fine whitish peripheral scale distributed on the back, shoulders, chest, arms, and legs. Skin biopsy was consistent with PLC. After minimal improvement with systemic and topical corticosteroids, oral upadacitinib 15 mg once daily was started. Following 1 month of treatment with oral upadacitinib, the complete remission of PLC was observed. Thus, upadacitinib as a Janus kinase (JAK) inhibitor may be a novel treatment for PLC. However, more studies are needed to explore the efficacy and safety of JAK inhibitors for lichenoid disorders, such as PLC and others.

## 1. Introduction

Pityriasis lichenoides chronica (PLC) is a chronic cutaneous disorder, which generally presents with pruritic or asymptomatic erythematous or hypopigmented papules and plaques. The etiology of PLC is unknown, but it is thought to be a proliferation of T‐cells in response to an antigen or infection leading to an inflammatory response. This disorder exists on the continuum with pityriasis lichenoides et varioliformis acuta (PLEVA), which presents acutely with crops of macules and papules that can become hemorrhagic and necrotic and eventually resolve with varioliform scars. Histologically, PLC is characterized by CD4^+^ lymphocytes as lymphocytic infiltrates in the dermis [1]. There are no US Food and Drug Administration (FDA) approved treatments for PLC, with most treatments used being antibiotic therapy with erythromycin, azithromycin, tetracyclines, and narrowband ultraviolet B often yielding variable and unpredictable results. This study presents a 62‐year‐old female with PLC who had a 4‐month history of erythematous nonpainful eruption, involving her trunk and extremities that resolved after 1 month of treatment using upadacitinib with virtually no postinflammatory hyperpigmentation.

PLC along with other pityriasis lichenoides (PL) are known for their resistance to conventional therapies making combination therapies a common choice for management [2]. Moreover, PLC can spontaneously resolve and regress over the course of several weeks and is known to have remissions where the entire course of the disease can take several years [3]; in this case, monotherapy with upadacitinib resulted in the improvement of symptoms and complete resolution of the rash, with continued ongoing remission well after a year at follow‐up.

This report provides preliminary evidence that upadacitinib, a Janus kinase (JAK) inhibitor, can be used as an effective and alternative treatment for treatment‐resistant PLC, achieving long‐term clearance, and highlights mechanism directed therapy for lichenoid dermatoses.

## 2. Case Presentation

**FIGURE 1:**
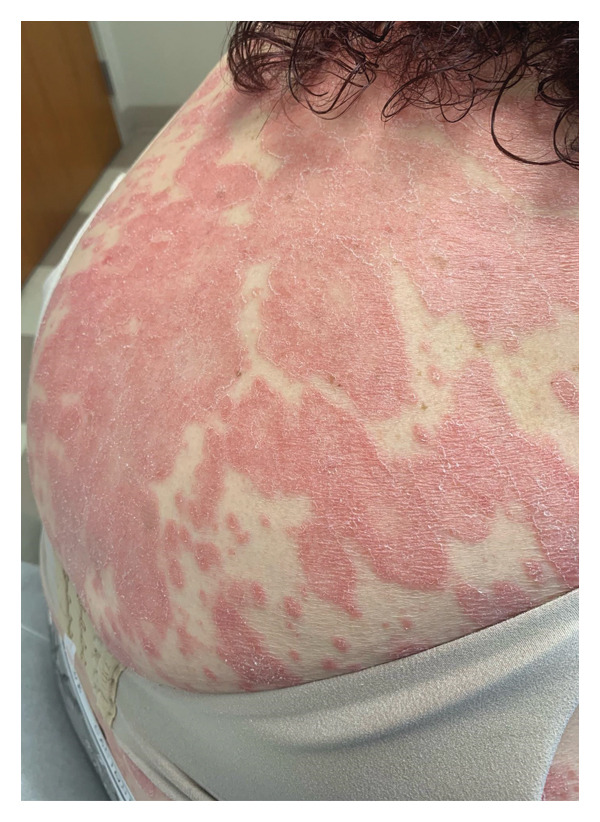
Initial presentation with erythematous annular and polycyclic papules and plaques extended over the back and shoulders.

A 62‐year‐old Caucasian female presented to clinic with a 4‐month history of diffuse erythematous nonpainful eruption, involving her back, shoulders, chest, arms and legs (Figure 1). Her medical history included anxiety disorder, arthritis, and atrial fibrillation, controlled by hydroxyzine, celebrex, and toprol, respectively. She reported no family history of dermatological disorders, no recent travel, infections, or changes in medications.

**FIGURE 2:**
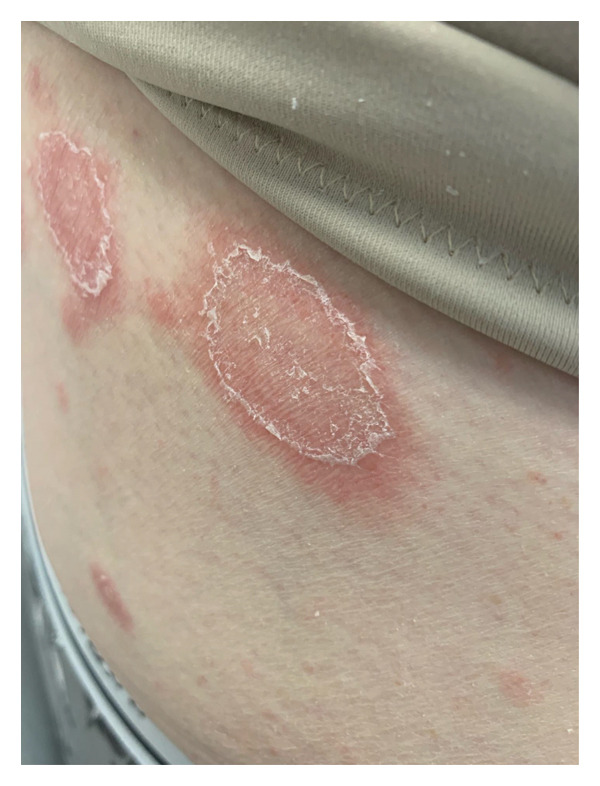
Erythematous plaques with a fine white peripheral scale on the lower back.

She was initially prescribed systemic as well as topical corticosteroids with limited efficacy. On examination, there were well‐demarcated erythematous annular and polycyclic papules and plaques with fine whitish peripheral scale distributed on the back, shoulders, chest, arms, and legs (Figure 2). Our differential diagnosis included PLC, psoriasis, lichen planus, and erythema annulare centrifugum.

**FIGURE 3:**
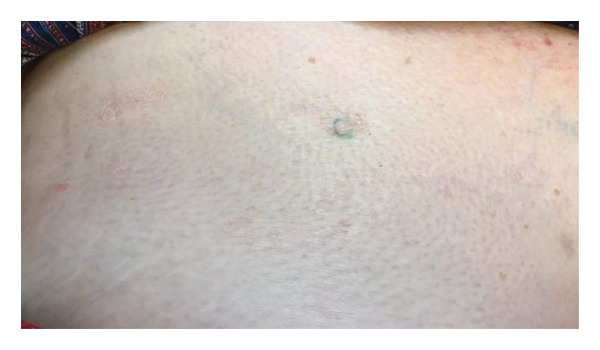
Clinical image of a punch biopsy site on right medial superior chest.

**FIGURE 4:**
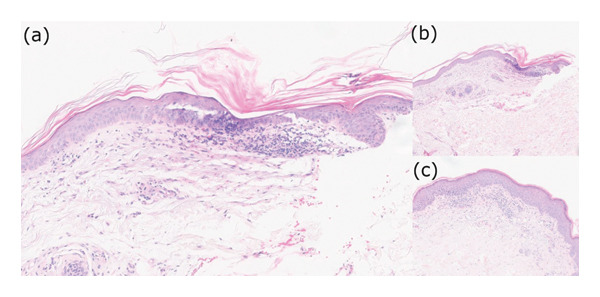
Histopathological section showing hyperkeratosis, parakeratosis, and lichenoid infiltrates with necrotic keratinocytes at the dermal–epidermal junction (H&E: (a) 20x magnification, (b) 10x magnification, and (c) 20x magnification).

Punch biopsy was performed (Figure 3), and the pathology report was consistent with PLC and was significant for hyperkeratosis, focal parakeratosis, and mild spongiosis. There is also lichenoid infiltrate of lymphocytes at the dermal–epidermal junction, and rare vacuoles and necrotic keratinocytes are identified (Figure 4).

**FIGURE 5:**
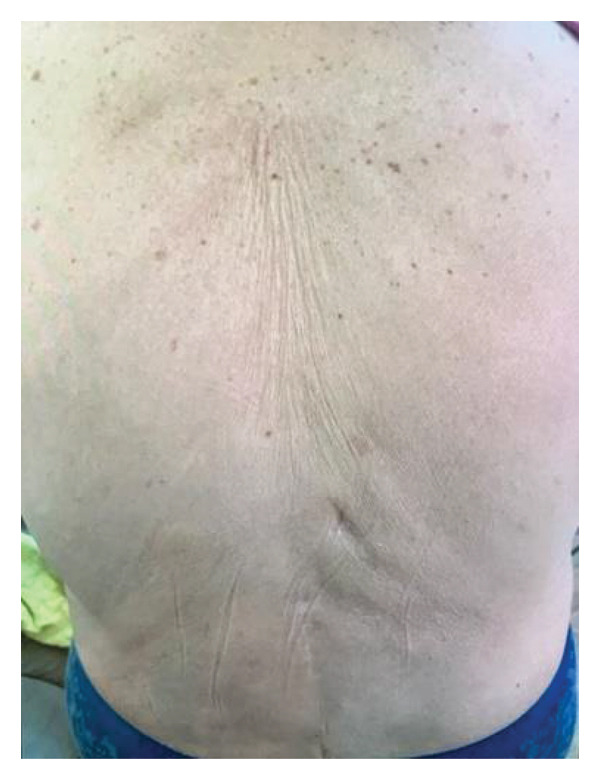
Interim clearance of pityriasis lichenoides chronica on regimen daily upadacitinib 15 mg over a 1‐month period.

These histopathological findings, patient disease history, and the lack of response to prior corticosteroid treatment along with emerging evidence for JAK inhibitor’s efficacy for lichenoid disorders made upadacitinib the choice of treatment. After obtaining fully informed consent and ensuring lack of contraindications, she was prescribed upadacitinib 15 mg once daily. She was seen again on telehealth after 1 month of treatment, at which time she showed complete resolution of the rash with no scarring and only mild postinflammatory pigmentation (Figure 5). She remained on upadacitinib 15 mg daily for another month, after which time she discontinued the medication and has continued in remission for over a year.

## 3. Discussion

PLC exists on the disease spectrum with the acute form of PLEVA and is thought to be a response to foreign antigens such as certain infections (Epstein–Barr virus, hepatitis C, and human immunodeficiency virus [4]) or medications (infliximab and adalimumab [5]), which results in T‐cell clonality and proliferation. Whether PLC is lymphoproliferative disorder with uncertain malignant potential is debated heavily in literature [6]. PL affects patients from all age groups and races with variable presentations. Additionally, the clinical course of PL can be diverse, and PLC is known to have spontaneous resolution and remissions over long periods of time and lesions often leave behind hyper‐ or hypopigmented macules [3]. The pathology of both PLC and PLEVA is distinctive with PLC containing lesional T‐cell infiltrates with a predominance of CD4^+^ cells [1]. In comparison to PLEVA, PLC has mild epidermal lymphocytic exocytosis, parakeratosis, keratinocyte necrosis, and lymphocytic infiltrate in the dermis [5, 6].

There are no FDA‐approved treatments for PLC with most treatments based on low level evidence such as case reports. Some common treatments include topical corticosteroids, systemic antibiotic (erythromycin, azithromycin, and tetracyclines), low‐dose methotrexate, and phototherapy, leading to inconsistent results. For this case, upadacitinib was used off‐label, leading to fast and complete resolution of the rash. This case is unlikely to have resolved spontaneously due to previous resistance to treatment and rapid response to upadacitinib within a month.

Upadacitinib is a reversible inhibitor of the JAK signal transducer and activator of JAK/STAT transcription signaling pathway, which inhibits the phosphorylation of downstream effector proteins and their subsequent cytokine signaling [7]. Upadacitinib is known to downregulate multiple potent effector cytokines, notably IFN‐γ, IL 4, IL 17, and IL 23 [8, 9], among others, thus leading to inhibiting several T helper (Th) cell differentiation pathways, such as Th1 and Th2, as well as Th17 [10].

Although it is unknown which Th cell phenotype is activated primarily in PLC, we postulate it is likely a Th1 driven inflammatory process, displaying a similar imbalance of Th1/Th2 phenotypes seen in other lichenoid conditions, such as lichen planus [11].

In conclusion, this patient quickly improved with oral upadacitinib as complete remission of PLC was observed after 1 month of treatment. In similar literature, upadacitinib has been described to successfully treat refractory PLC with mild hyperpigmentation at 3 months or in a similar case with significant remission at 6 months [12]. This case demonstrates successful treatment of PLC with upadacitinib as a primary monotherapy with complete resolution at 1 month with ongoing remission over a year after discontinuation. Moreover, PLC is known to spontaneously relapse with a disease course that can last years, so the patient will be followed long term to identify any possible relapses in future visits.

In dermatology, there is a significant unmet need of treatment options which can safely and effectively inhibit Th1‐driven inflammatory processes of lichenoid diseases. This case report, along with others springing up in literature, highlights the potential role JAK inhibitors may play in the treatment of PLC as well as other lichenoid dermatoses, such as cutaneous and mucous membrane lichen planus and lichen planopilaris [13].

## Funding

No funding was received for this research.

## Consent

The authors obtained written consent from the patient for their photographs and medical information to be published in print and online and with the understanding that this information may be publicly available. Patient consent forms were not provided to the journal but are retained by the authors. Patient information cannot be identified from the photos provided to the journal.

## Conflicts of Interest

The authors declare no conflicts of interest.

## Data Availability

The data that support the findings of this study are available on request from the corresponding author. The data are not publicly available due to privacy or ethical restrictions.
